# Research on vehicle detection based on improved YOLOX_S

**DOI:** 10.1038/s41598-023-50306-x

**Published:** 2023-12-27

**Authors:** Zhihai Liu, Wenyu Han, Hao Xu, Kesong Gong, Qingliang Zeng, Xieguang Zhao

**Affiliations:** 1 College of Transportation, Shandong University of Science and Technology, Qingdao, 266590 China; 2 College of Mechanical and Electronic Engineering, Shandong University of Science and Technology, Qingdao, 266590 China; 3 College of Intelligent Equipment, Shandong University of Science and Technology, Taian, 271001 China

**Keywords:** Computer science, Information technology

## Abstract

Aiming at the problem of easy misdetection and omission of small targets of long-distance vehicles in detecting vehicles in traffic scenes, an improved YOLOX_S detection model is proposed. Firstly, the redundant part of the original YOLOX_S network structure is clipped using the model compression strategy, which improves the model inference speed while maintaining the detection accuracy; secondly, the Resunit_CA structure is constructed by incorporating the coordinate attention module in the residual structure, which reduces the loss of feature information and improves the attention to the small target features; thirdly, in order to obtain richer small target features, the PAFPN structure tail to add an adaptive feature fusion module, which improves the model detection accuracy; finally, the loss function is optimized in the decoupled head structure, and the Focal Loss loss function is used to alleviate the problem of uneven distribution of positive and negative samples. The experimental results show that compared with the original YOLOX_S model, the improved model proposed in this paper achieves an average detection accuracy of 77.19% on this experimental dataset. However, the detection speed decreases to 29.73 fps, which is still a large room for improvement in detection in real-time. According to the visualization experimental results, it can be seen that the improved model effectively alleviates the problems of small-target missed detection and multi-target occlusion.

## Introduction

In recent years, as Chinese traffic networks have expanded and public demand for travel has increased, car ownership has risen steadily, leading to road safety and traffic control issues, frequent traffic accidents, and severe traffic jams. This situation can lead to secondary injuries if not handled promptly. These issues will affect traffic flow and the vehicle's stability and comfort. It is also a significant threat to road safety. Vehicles are one of the critical elements in any traffic scenario to maintain the balance and safety of the system, and vehicle detection is the first step and critical component of a traffic event detection system; hence, achieving more accurate vehicle target detection is of significant research value. With the significant development of intelligent traffic systems and machine vision and the increase in the degree of video surveillance coverage, the research on intelligent traffic monitoring and intelligent traffic accident detection continues to increase, with breakthroughs in multi-target and small-target detection techniques for urban road and highway traffic scenes. Intelligent detection of vehicle targets using graphics processing and video analytics can provide the basis for subsequent vehicle tracking and traffic event detection. It can also avoid problems such as time-consuming surveillance and statistical errors due to manual or traditional sensor detection. Due to the influence of different weather, light intensity, the complex background of the traffic scene, and the size of the moving target changing at any time, the vehicle detection effect will be disturbed to a certain extent in the actual traffic monitoring scene. Therefore, this paper uses deep learning-based algorithms to improve vehicle detection accuracy to investigate vehicle detection techniques in traffic scenarios.

The research direction of vehicle detection algorithms is divided into traditional detection algorithms and deep learning-based detection algorithms. Traditional vehicle detection algorithms pay more attention to feature extraction and need to rely on manual feature extraction to construct the detection model, whose main steps are divided into candidate region selection, feature extraction, and candidate box classification. This algorithm usually uses various ways to extract local features of the vehicle for identification and detection, such as HOG, SIFT, and DPM. For example, to improve the accuracy of vehicle detection, Wang Zhangu and others^1^ extracted local and global features of vehicles in infrared images using HOG features and LBP feature fusion, which improved the detection accuracy and speed compared to the traditional sensor detection methods; Kenan Mu and others^2^ proposed a vehicle detection method combining the SIFT algorithm and computer vision, which compared the images of the two frames before and after to automatically mark the location of the target features according to the geometric relationship, which improved the accuracy of the detection algorithm, but the method is only suitable for traffic scenes without pedestrians or non-motorized vehicles; Dongbing Zhang^3^ proposed an improved DPM method. The method first extracted the channel information of the color space transformed image and trained the DPM of each channel, then used the adaptive fusion method to obtain the color fusion DPM, and finally searched the region beyond the threshold through the sliding window and designated this part of the region as the vehicle target. Experiments verified the effectiveness of the method, and the false detection rate was reduced. These algorithms need to rely on manual use under specific scenario conditions, cumbersome detection steps, which are not conducive to the implementation of large-scale rapid detection, and unstable models, resulting in insufficient overall learning of vehicles.

**Figure 1:**
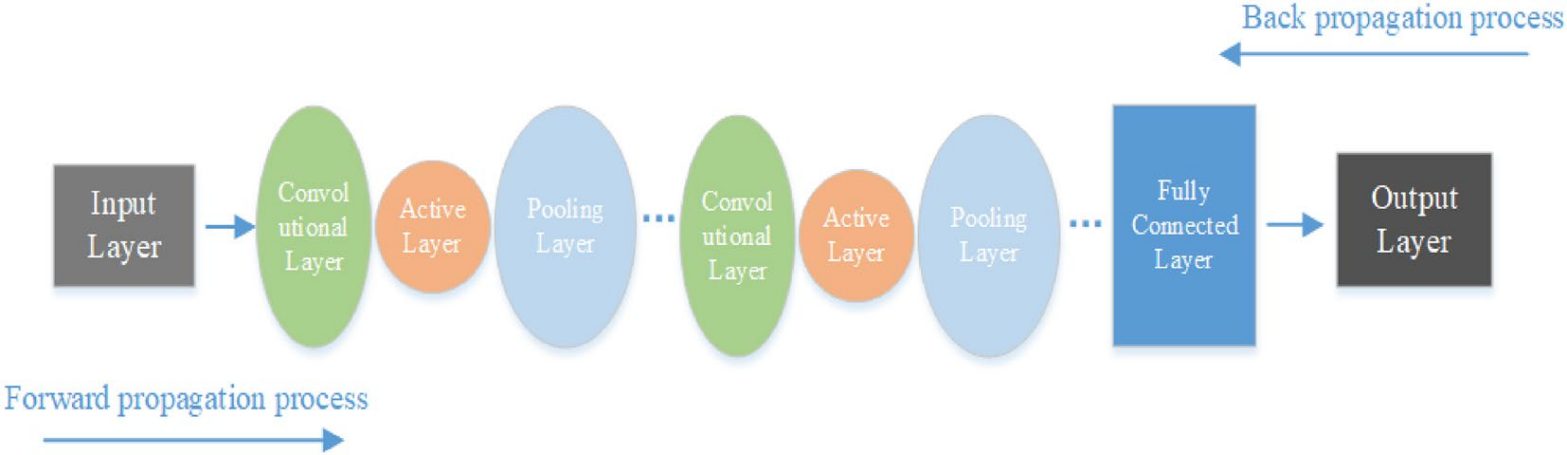
Convolutional Neural Network structure.

Compared with traditional detection methods, deep learning-based vehicle detection techniques use Convolutional Neural Network (CNN) to learn the overall information, with better model generalization and higher robustness. The CNN can reduce the amount of manual testing and lower labor costs. In forward propagation, the convolutional layer and the pooled layer process the image multiple times to obtain the feature vector. The feature vector is classified and recognized after passing into the fully connected layer, and the prediction result is compared with the recognition result. If the two do not match, backpropagation is performed: the error between the output and the prediction is fed back one layer at a time in the opposite direction, the error for each layer is calculated, and the weights are updated. The structure of the convolutional neural network CNN is shown in Fig. 1.

Deep learning-based target detection methods not reliant on manual feature extraction can be classified into two categories at this stage. One category is the two-stage target detection methods represented by R-CNN^4^, Fast R-CNN^5^, Faster R-CNN^6^, and Mask RCNN^7^. In these methods, the first class of convolutional neural nets regresses and locates the candidate frames after generating the target candidate frames. Then, the second class of convolutional neural nets completes the classification of the candidate frames using classifications. Therefore, the two-stage detection algorithm has a high detection accuracy, but the detection speed needs to be higher, which cannot meet the requirements of fast detection in real scenes. The other category is the one-stage target detection methods represented by RetinaNet^8^, SSD^9^, and YOLO^10^ series algorithms. These methods do not need to generate pre-selected boxes and can directly use convolutional neural networks for end-to-end processing, generating multiple bounding boxes in the image, predicting the class probability of each box, and performing the classification and regression tasks simultaneously. Regarding detection speed, one-stage target detection methods outperform two-stage target detection methods, but the detection accuracy is lower. Current research on detection technology focuses mainly on single-stage detection algorithms and continuously optimizes and improves detection algorithms for the problem of detection accuracy. Zhang Luyang et al.^11^ addressed the problem of low efficiency of vehicle detection in real traffic scenes by introducing octal convolution into the RetinaNet network to optimize the learning effect of detailed features. They introduced the WFPN structure to improve further the feature fusion to trade off the propagation and fusion between features of different scales. The experimental results showed that the improved model can be better applied to the needs of real scene detection. Chen Zhichao et al.^12^ used the MobileNetv2 structure as the backbone network to reduce the number of model parameters. The channel attention module was introduced to improve the detection accuracy, and the bottom-up feature fusion network was constructed using the inverse convolution module. The experimental results show that the detection accuracy and detection speed are greatly improved. The YOLO-based target detection algorithm detects fast and can realize end-to-end detection. The model has good robustness and generalization ability, which is applied to the research of vehicle detection. For example, Tianyu Tang^13^ applied YOLOv2, which is an improved version of YOLO to UAV vehicle detection, and further improved the detection accuracy on a real-time basis; Lecheng Ouyang et al.^14^ trained the vehicle detection model based on the YOLOv3 algorithm, and the experimental results showed that compared with the traditional target detection algorithms, the method had advantages in detection accuracy and speed; Lin et al.^15^ used the YOLOv4 model as a detector to realize directional vehicle detection in aerial images, and the experimental results showed that the method improved the detection speed by 25% while maintaining high detection accuracy. These empirical studies collectively proved the successful application of YOLO series algorithms in vehicle detection and provided references for the development of vehicle detection technology. With the continuous deepening of the research on YOLO series algorithms, algorithms such as YOLOv5, YOLOX, and YOLOv7 have appeared, and researchers have achieved a large number of results in the field of vehicle detection research based on the YOLO model with improved network structure. For example, Ge et al.^16^ based on the YOLOv3-tiny model, replaced the feature extraction network with lightly weighted networks such as DarkNet-19 and ResNet-18 and used the K-means algorithm to achieve anchor frame clustering, and the experimental results showed that the average accuracy was improved by 14.09% and the detection speed was improved by 13 fps; Zhang Yong et al.^17^ used the MobileNetv3 structure to lighten and improve YOLOv4, which achieved the association of features at different levels through a multi-scale feature fusion method and introduced a coordinated attention mechanism to improve the attention to the target. The detection accuracy was improved to 85.79%, reducing the missed detection rate; Dong Xudong et al.^18^ proposed a lightweight YOLOv5 model by introducing the C3Ghost and Ghost modules in its neck network, introducing the CBAM attention mechanism in the backbone network, and replacing the loss function with the CIOU, and after experimental verification, the improved model improved the detection accuracy by 3.2% and reduced the model parameters by 19.37% compared with the original model; Zhang Yuan et al.^19^ proposed an improved YOLOv7-RAR algorithm, which utilized the Res3Unit structure to reconfigure the original YOLOv7 backbone network, added the ACmix attention module after the SPPCSPC layer, and used the RFLA module to improve the sensory field. Experiments showed that compared with the original algorithm, the detection accuracy of this algorithm was improved by 2.4%, and it could be better applied to vehicle detection.

After the YOLOX algorithm was proposed in 2021, the researchers used it to target recognition and detection tasks in various fields. They made a significant breakthrough in the detection performance by optimizing its network structure. For example, Wang Xin et al.^20^ improved the YOLOX model for the problem that it is not easy to detect small targets with complex backgrounds in UAV aerial photography by incorporating the Ultra-Lightweight Quantum Spatial Attention Module (ULSAM) as well as optimizing the loss function in the PAN structure. The experimental results showed that the detection accuracy was improved by 8%. Wang Jingjing et al.^21^ used an autonomous cooperative mechanism to cut out redundant structures based on the original YOLOX model, strengthened the target features through a lightweight self-attention mechanism, and introduced a segmented focus loss function to weigh the sample weights. The experimental results showed that the enhanced model improved the detection accuracy over the original model; Hong Wang and others^22^ proposed a multilevel fine-grained YOLOX detection algorithm by introducing a ResCoT module in the PAFPN structure to expand the receptive field region and adding a normalized attention mechanism to the CSPLayer structure and finally using comparative experiments to determine the optimal parameter values of the loss function, and the improved algorithm improved the detection accuracy by 2.90%; Yi Kefu et al.^23^ experimented with the detection algorithms using a training strategy of data domain migration for the problem of small and multi-target detection occlusion under low-light conditions at night. They added a coordinate attention module to the effective feature layer of the YOLOX backbone network, introduced a feature fusion module to the FPN structure, and combined the CIOU with a loss of confidence function. The average accuracy was improved by 5.9%; He Qiyi et al.^24^ proposed a lightweight ShuffYOLOX model by using ShuffDet instead of CSPDark53 network and introducing an ECA attention module in the PAFPN network, which improves the detection accuracy while lightweighting the model; Zhu Chao et al.^25^ introduced the ECA attention module in the feature extraction network and combined the training strategies of cross-domain migration and intra-domain migration, which resulted in an improved model with a detection accuracy of 95.65%; Liu Changhong et al.^26^ incorporated lightweight operators into the feature extraction structure to adaptively extract channel features, and then introduced the CBAM attention module in the PAFPN network and designed the decoupled head architecture to reduce gradient vanishing to accelerate model convergence, which finally resulted in a 1.47% improvement in model detection accuracy.These studies have demonstrated that it is possible to improve the feature extraction network by optimizing the network structure of YOLOX, which leads to improved target detection accuracy while ensuring that it meets the requirements of practical applications.

In this context, this paper optimized and improved the network structure of the original YOLOX model to improve the problems of low detection accuracy of small targets and omission of multi-target occlusion when detecting vehicles by using traffic monitoring. The main research work is as follows:

1. The complex structure of the YOLOX network with more redundant information between pixels leads to slow model inference and affects the detection speed. Therefore, the unimportant channels in the network structure are clipped by adding a sparse factor to the BN layer;
2. Incorporating the Coordinate Attention module into the YOLOX backbone network helps the network to locate target features more quickly and accurately in a large amount of feature information and enhances the feature extraction capability;
3. The implementation of deep and shallow feature fusion at different scales in YOLOX's neck network will result in under-utilization of feature information, so the adaptive feature fusion module is added after the PAFPN network to adjust the proportion of positive and negative samples using weights to improve the feature extraction capability;
4. Since the imbalance of positive and negative samples leads to the low detection accuracy of YOLOX, the Focal loss function is used instead of the SiLU loss function in the decoupled head structure to improve the convergence speed, alleviate the problem of low detection accuracy of difficult-to-classify samples, and enhance the ability to learn small target features.

## YOLOX

**Figure 2:**
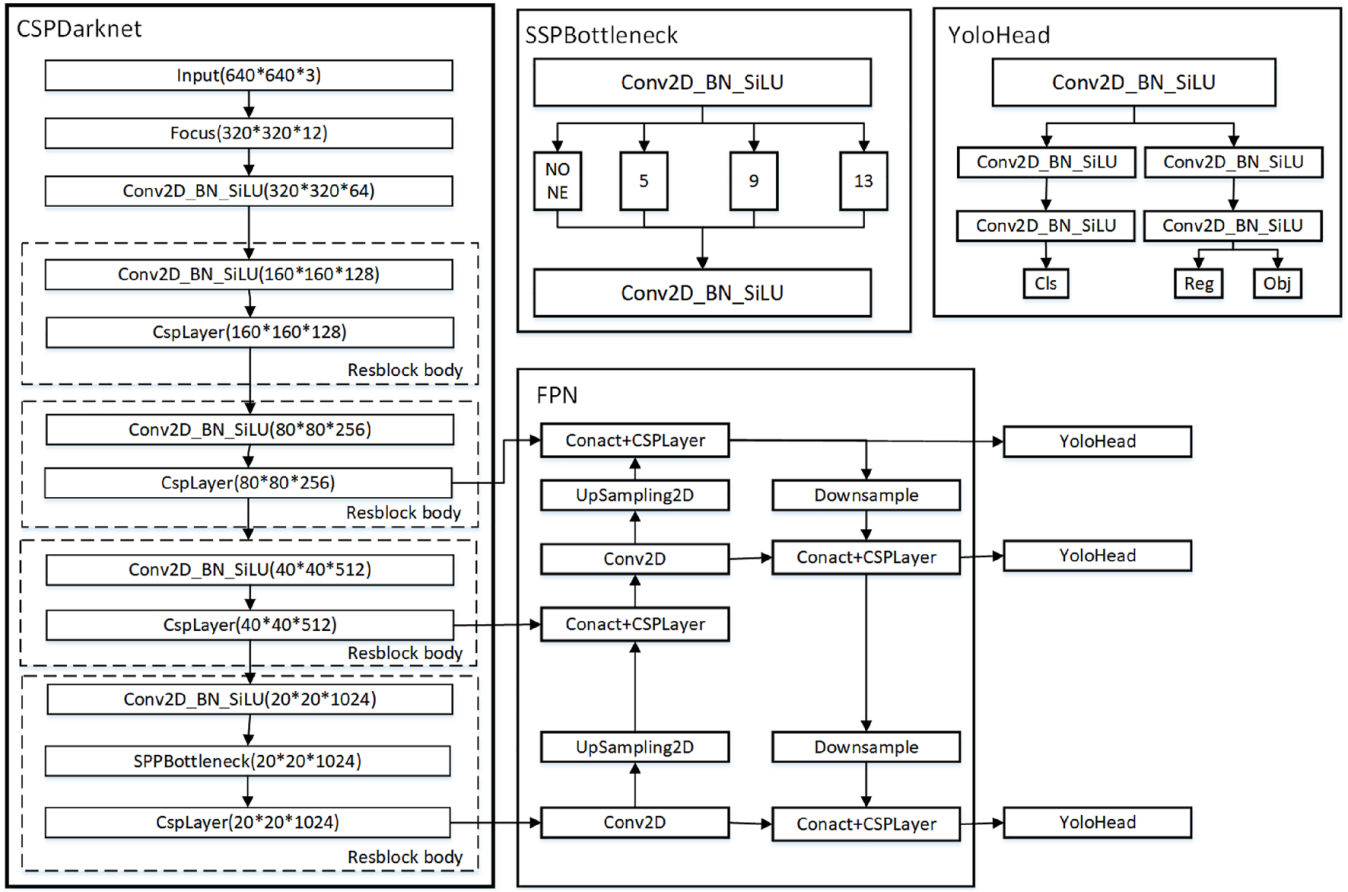
Schematic diagram of YOLOX network structure.

The network structure of the YOLOX algorithm mainly consists of four parts: the input, the backbone feature extraction network, which outputs adequate feature information; the neck structure, which uses a feature pyramid for multi-scale fusion to improve feature extraction; and the prediction layer with a decoupling head (YOLOHead), and the specific details of the network structure are shown in Fig. 2. The YOLOX algorithm model is designed to improve on the YOLOv3, YOLOv4, and YOLOv5 models by adding the MixUp data enhancement method to the Mosaic data enhancement on the input side to enrich the data set; SiLU activation functions replace the inference modules in the backbone and neck structures; Adding Decoupled Head, Anchor Free mechanism and refined allocation strategy SimOTA at the output layer directly associates the actual frame with the predicted frame, which reduces the amount of model computation and therefore its detection speed is faster.

**Figure 3:**
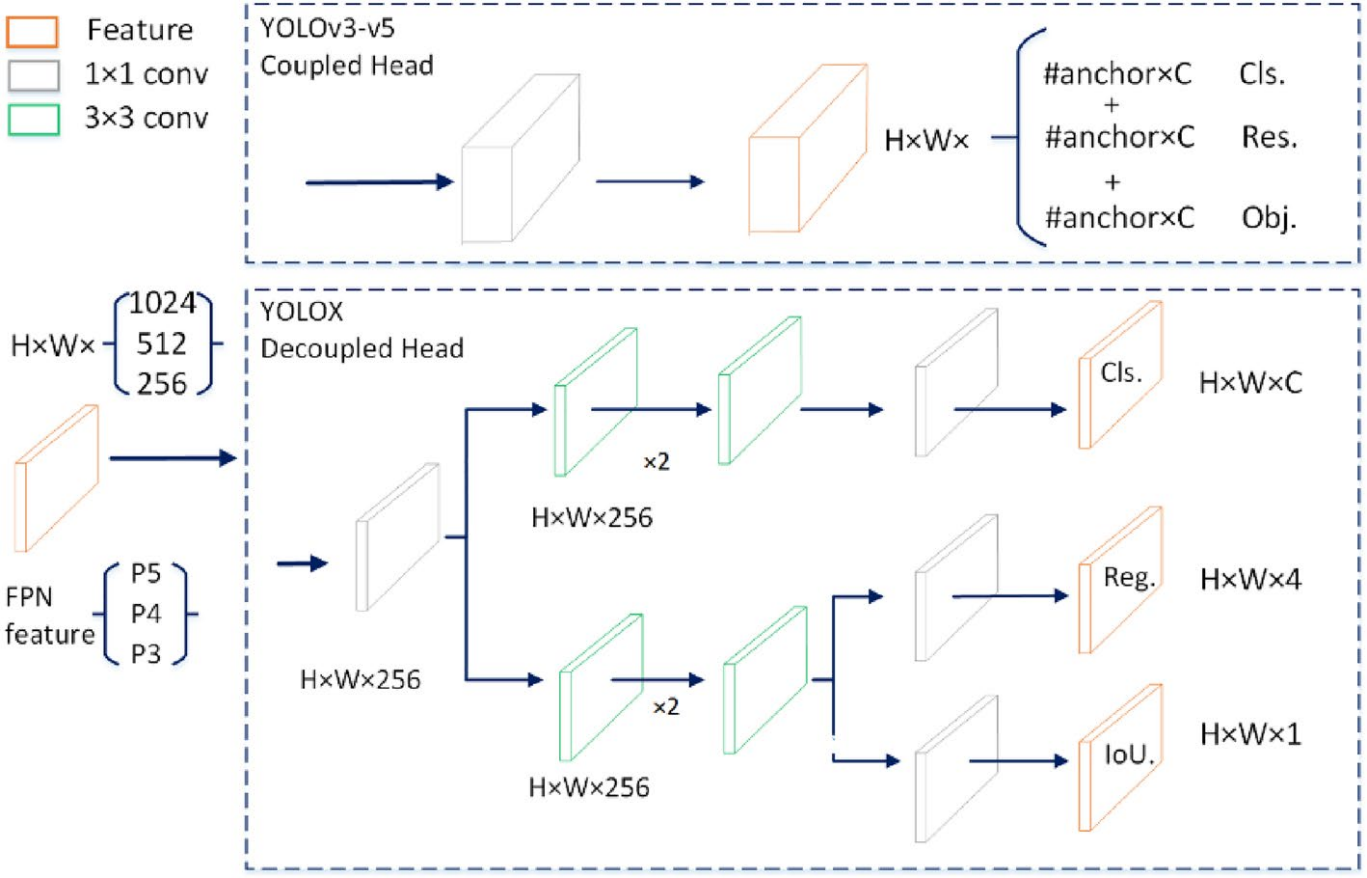
Schematic diagram of the Decoupled Head structure.

The significant improvement of the YOLOX model over the previous models of the YOLO algorithms is that the coupled detection head is changed to a decoupled head structure in the Prediction layer (head part). The structure is shown schematically in Fig. 3. In the Coupled Head, the neck part of the output feature prediction process is in the same convolutional layer, and the use of a fully connected layer or convolutional layer on the input feature map to directly generate the information of the target category and localization, resulting in a conflict between the classification and regression tasks, and the coupling process will produce a large number of parameters prone to overfitting. In the Decoupled Head, a convolution normalization and activation function is first used to perform a dimensionality reduction operation on the output feature maps of the Neck part, keeping the number of channels the same. Then, the classification and regression tasks are split into two branches simultaneously: The first branch is the obtaining of the classification prediction result cls (categories) after two convolution normalization and activation functions; The second branch produces the regression prediction result obj (localization) for the detection frame and the judgment result reg (IOU) for the presence or absence of a target at the feature point, again after two convolutional normalizations and activation functions. Finally, the prediction results of three different scales are spliced to get the predicted feature information under that dimension.

**Figure 4:**
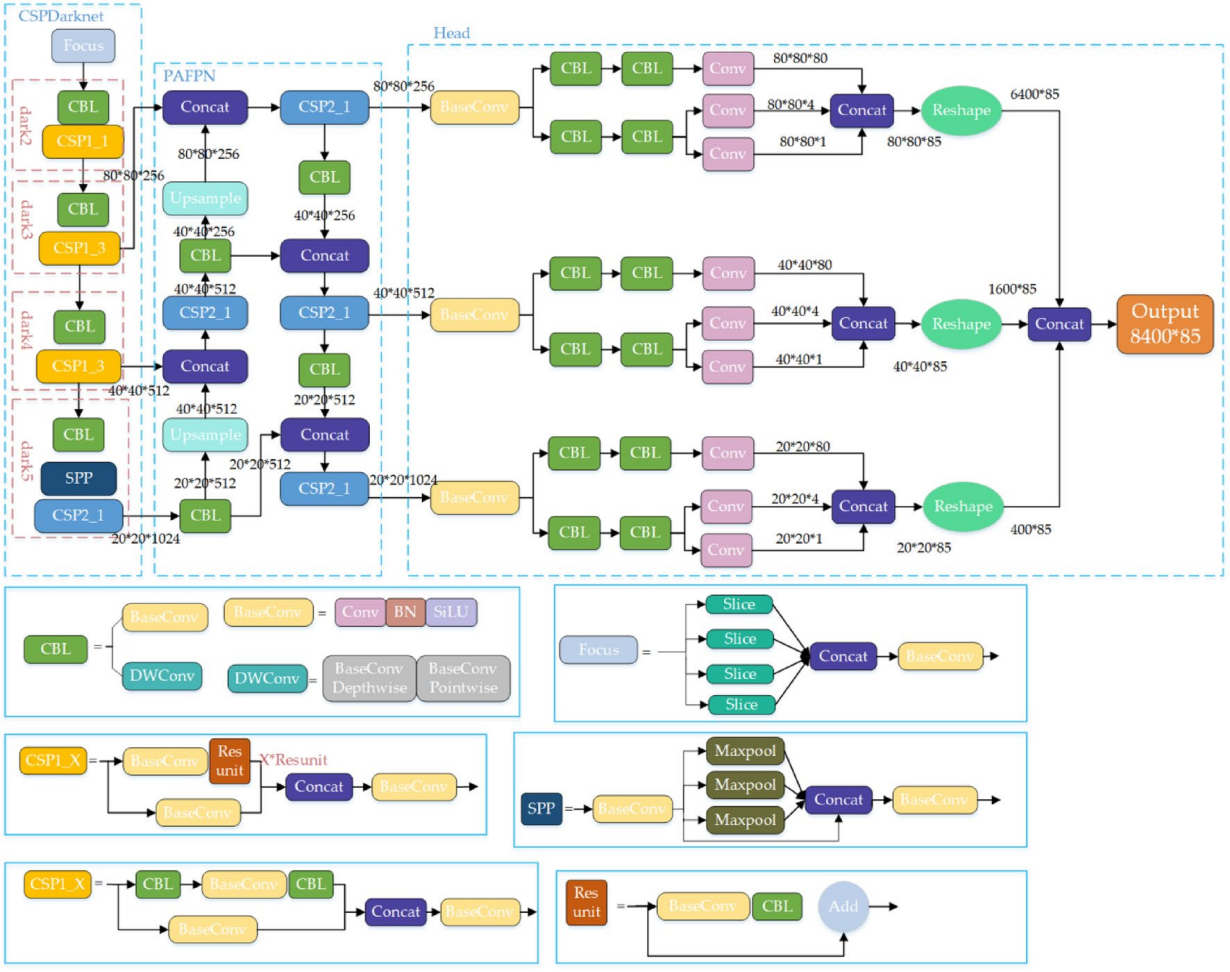
Schematic diagram of the YOLOX_S network structure.

According to the scaling principle, the YOLOX algorithm designs five kinds of standard networks, such as YOLOX_S/M/L/X, and two kinds of lightweight networks, such as YOLOX-Nano and YOLOX-Tiny. In this paper, we adopt the YOLOX_S model, which has fewer number of model parameters, and the structure of its network is shown in Fig. 4. In the figure, the blue solid box indicates the specific architecture of each module in the network structure, “Add” means the overlay operation, and “Concat” means the dimensional splicing operation.

The input side is improved based on Mosaic data enhancement, which merges four simultaneously read images by randomly scaling, cropping, and flipping and detects small targets. MixUp data enhancement combines two images using a specific parameter to enhance the ability to extract detailed features and detect small tarfvgets.

**Figure 5:**
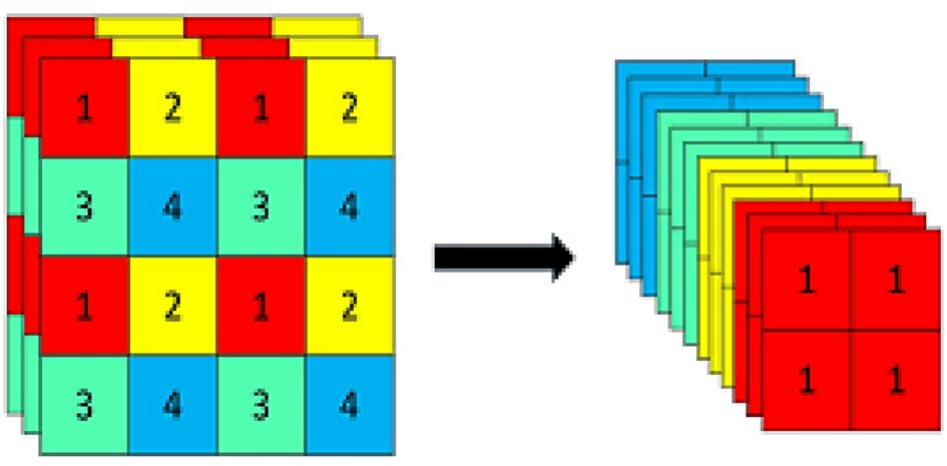
Focus slicing operation.

The backbone network (feature extraction network) mainly comprises the CSPDarknet structure of YOLOv5 and the Focus structure. The Focus module splits the input high-resolution image into four low-resolution images by slicing, shown in Fig. 5. It performs channel splicing, which converts the image information into the channel information and prevents the loss of small target feature information caused by downsampling. After CBL convolution operation of the residual network and SPP structure for different feature layers using maximum pooling operation for feature extraction.

The Neck structure (multi-scale feature fusion network) follows the path fusion pyramid network structure PAFPN of YOLOv4, and the central role is to enhance feature extraction using the CSP structure. The FPN network performs the up-sampling operation on the deep features of the effective feature layer input from the backbone network, merges and passes them with the shallow features, and then uses the path aggregation network PAN to perform the down-sampling operation on the shallow features and then merges them with the deep features to obtain the feature information at three scales.

The Prediction layer uses three decoupling heads to predict the three feature layers with different scales in three branches, and the decoupling heads are divided into two parts to achieve classification and regression, respectively. Integrating the three branches information in the final prediction can effectively accelerate the convergence speed of the model and improve the detection accuracy. Then, the anchorless mechanism is used to compare the target and actual frames to determine the gap between them. Finally, the positive samples are dynamically allocated by the SimOTA method.

The above designs significantly increase the upper limit of model detection performance. While retaining the advantages of the previous YOLO series algorithms, methods such as decoupling head, anchorless frame mechanism, and new data enhancement methods are added to enhance the feature extraction capability of the model.

**Figure 6:**
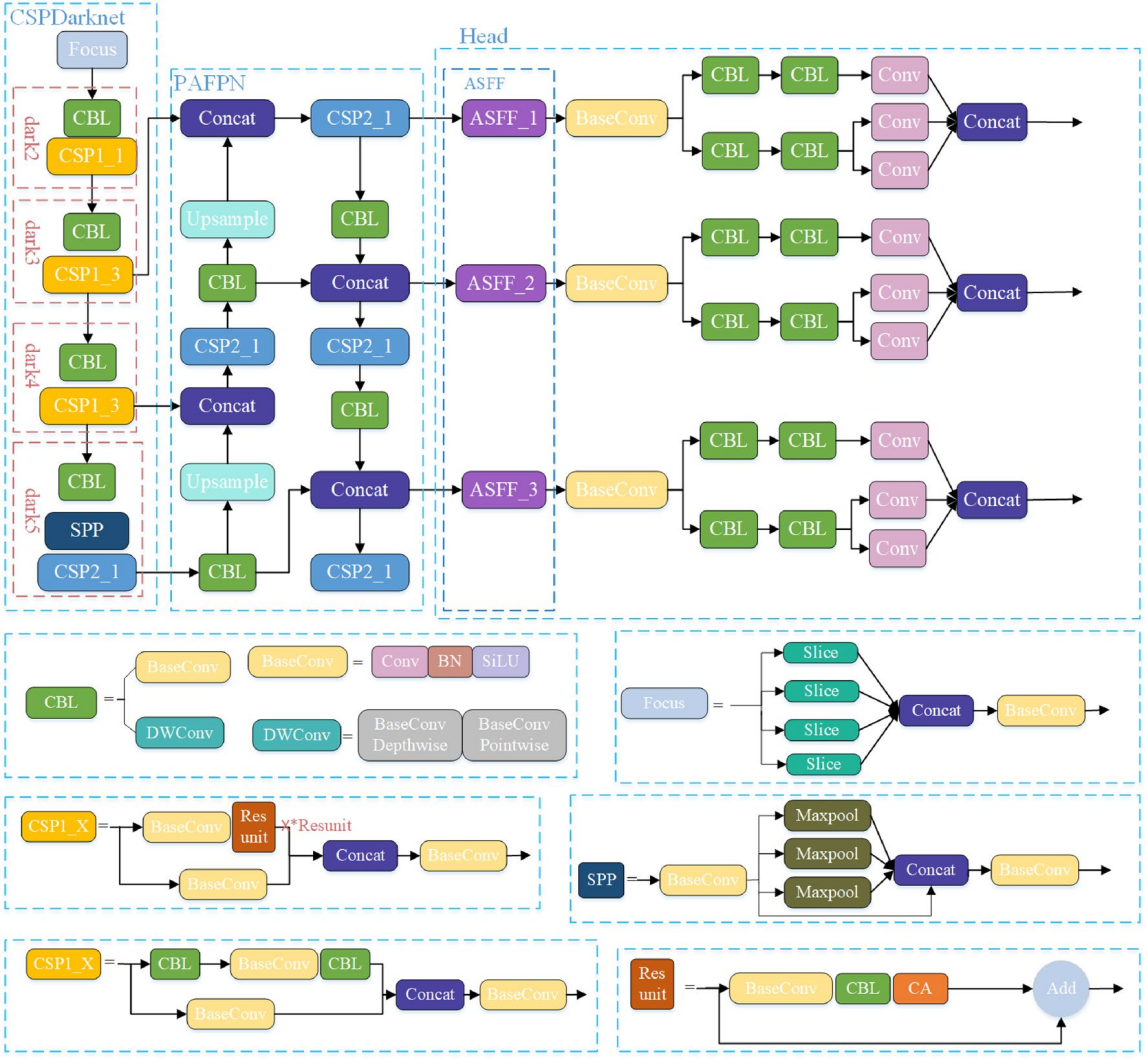
Improved YOLOX_S network structure.

Aiming at the problems of small target detection accuracy and multi-target detection leakage in vehicle detection, this paper improves the original YOLOX model network structure as follows: In this paper, firstly, the channel pruning method is used to simplify the YOLOX network model, and the number of parameters is reduced to improve the detection speed of the model. When training the model, sparse factors are introduced into the network's normalization layer, and each channel's contribution rate is calculated. According to the experimental verification of different pruning rates, the pruning rate with the optimal detection effect is selected to prune the channel with a low contribution rate, and then the model with the best detection performance after pruning is trained by fine-tuning the model; secondly, the Coordinate Attention module is selected to make full use of the spatial feature information and the channel feature information and to improve the ability of small target feature extraction. The CA module is placed after the CBL convolution operation of the residual structure, after the three adequate feature layers of the backbone network dark3, dark4, and dark5, as well as the above two positions simultaneously. The model is trained on the dataset of this paper to verify which placement position is the best, and the results show that the placement of the CA module in the residual structure is the best for the detection performance; thirdly, the adaptive feature fusion module is added behind the path aggregation feature pyramid network PAFPN, using the weight coefficients to increase the proportion of shallow features before inputting the three branches of the decoupling head for feature prediction to improve the ability of small target feature extraction; finally, the SiLU loss function of the classification prediction part of the three decoupling heads in the prediction layer is changed to the focal loss function to optimize the problem of sample imbalance. On the re-labeled dataset in this paper, the improved YOLOX model achieves a recognition accuracy of 77.19%, 4.86% higher than the original YOLOX model. The overall framework of the improved algorithm is shown in Fig. 6.

## Methods

### Model pruning

The YOLOX network structure is highly complex, and the backbone and detection structures contain many convolution operations. Training the model once will produce many redundant weight parameters and channels, affecting the convergence speed. Effective model compression methods can be used to simplify the network structure and reduce the computational complexity of the model, and commonly used model compression techniques mainly include knowledge distillation, shared weights, model pruning, and low-rank decomposition. Knowledge distillation refers to constructing a lightweight, small model (student model) and learning to migrate the supervisory information contained in the already trained model (teacher model) into the small model and train it to achieve better detection performance. Wenjun et al.^27^ standardize the activation map of each channel, minimize the asymmetry of the Kull Back-Leibler (KL) scatter between the activation maps of the two models, and propose a soft technique that makes the student model focus on mimicking regions with activation values, which ultimately leads to better detection results for the student model than the teacher model. Shared weighting refers to the use of the same convolutional kernel (weight matrix) for feature extraction at different locations of the image in CNN, which makes the local features translation invariant; Liao et al.^28^ used DBSCAN to divide the dataset into clusters, clustered the density information, and set each cluster of all data points to set the same weight parameter. The final model obtained achieved better experimental results. Low-rank decomposition refers to decomposing a high-dimensional parameter vector into several low-dimensional parameter vectors and approximating the original high-rank parameter vectors with the low-dimensional parameter vectors; Wang et al.^29^ proposed a neural network based on low-rank and grouped sparse tensor decomposition, where the tensor of each convolutional layer is decomposed into multiple subtensors, and the sum of the subtensors is used to approximate the original weight tensor. Model pruning is the process of cutting out redundant structures that have a small impact on the detection results to reduce the number of model parameters while maintaining the detection accuracy to improve the model inference speed. Wu et al.^30^ used the model pruning method to optimize the YOLOv4 model, and the test results showed that the model size of the pruned detection model was reduced by 231.52 MB, and the model inference time was reduced by 39.47%. Guo et al.^31^ conducted test experiments of pruning operation on three network structures, namely ResNet20, VGG, and DenseNet40. The results showed that the computation of the deep neural network was reduced by the pruning method and the detection performance was maintained. Therefore, in this paper, the method of network pruning is chosen to simplify YOLOX.

Model pruning is divided into structured pruning and unstructured pruning. Unstructured pruning cuts out neurons whose weights are below a threshold and sets the connection weights between them and other neurons to 0. The simplified model is trained iteratively each time to reduce the loss of accuracy caused by pruning. The advantage of unstructured pruning is that the algorithm is simple, and filling the unimportant weights with zeros can improve model overfitting. The disadvantage is that most deep learning frameworks cannot accelerate the computation of sparse matrices, and they must rely on specific hardware environments to achieve the effect of compression and acceleration. In contrast, structured pruning is a method for pruning the importance of filters or network structure layers, which is divided into filter pruning, channel pruning, and layer pruning. The advantages of structured pruning are that the original convolutional structure can be preserved, and model compression can be achieved without a specific hardware environment; the disadvantages are that its algorithm is complex, the network structure is pruned, and the change of input and output dimensions will cause some deviations. To better simplify the depth and width of the model, this paper adopts the channel pruning method to process the YOLOX_S network structure, and the model pruning is mainly divided into three processes: sparse training, pruning processing, and fine-tuning parameters.

In practice, too much pruning leads to a significant reduction in detection accuracy, so it is necessary to constantly adjust the selection of the appropriate thinning rate and pruning rate. The specific process of the current model pruning is first to add L1 paradigm regular terms to constrain its coefficients in the BN layer to complete the coefficients sparsification training and then select the redundant parts whose pruning rate has a negligible impact on the network detection results to be pruned, and finally fine-tune the model and iteratively repeat the above process in each layer of the structure, which will be able to complete the channel scaling to improve the speed of the model detection.

#### Sparsification training

In the YOLOX network, a Batch Normalization layer is added after each convolutional layer for normalization to speed up network training and convergence. The gamma coefficients of the BN layer are set to the scaling factor γ multiplied by the output of its corresponding channel, and the following normalization formula normalizes the output of each layer:

1 $${\tilde{\text{c}}} = \frac{{R - \overline{x}}}{{\sqrt {s^{2} + \varepsilon } }}$$

2 $$C = \gamma \cdot {\tilde{\text{c}}} + \beta$$

In the formula, R and C represent the result of the convolutional computation of the layer (the input of the normalization process) and the output of the normalization process, respectively; $\varepsilon$ is a minimal non-zero value such that the denominator is not 0; $\overline{x}$ and s^2^ denote the mean and variance of R, respectively; β is the normalization bias, and both parameters β and γ are learnable.

The model must be sparsely trained before pruning because the network structure has different weights for each parameter. Different sparsity rates need to be set to test its adaptability during training; the higher the sparsity rate, the faster the BN layer converges, which can lead to a severe drop in accuracy; the smaller the sparsity rate, the slower the sparsity process. The L1 regular term is combined in the objective function to sparsify the gamma parameters and increase the robustness of the model. The objective function (total loss function) is as follows:

3 $$L{\text{oss}} = \sum\limits_{(x,y)} {l(f(x,W),y) + \lambda \sum\limits_{\gamma \in \Gamma } {g(\gamma )} }$$

4 $$g(x) = \left| x \right|$$

In the formula, the item l(·) to the left of the plus sign is the original loss function; the item to the right is the regularization term (constraint); x and y represent the training data and labels, respectively;${\uplambda }$ are the compensating factors of the two terms, which can be adjusted according to the data set.

#### Pruning treatment and fine-tuning

**Figure 7:**
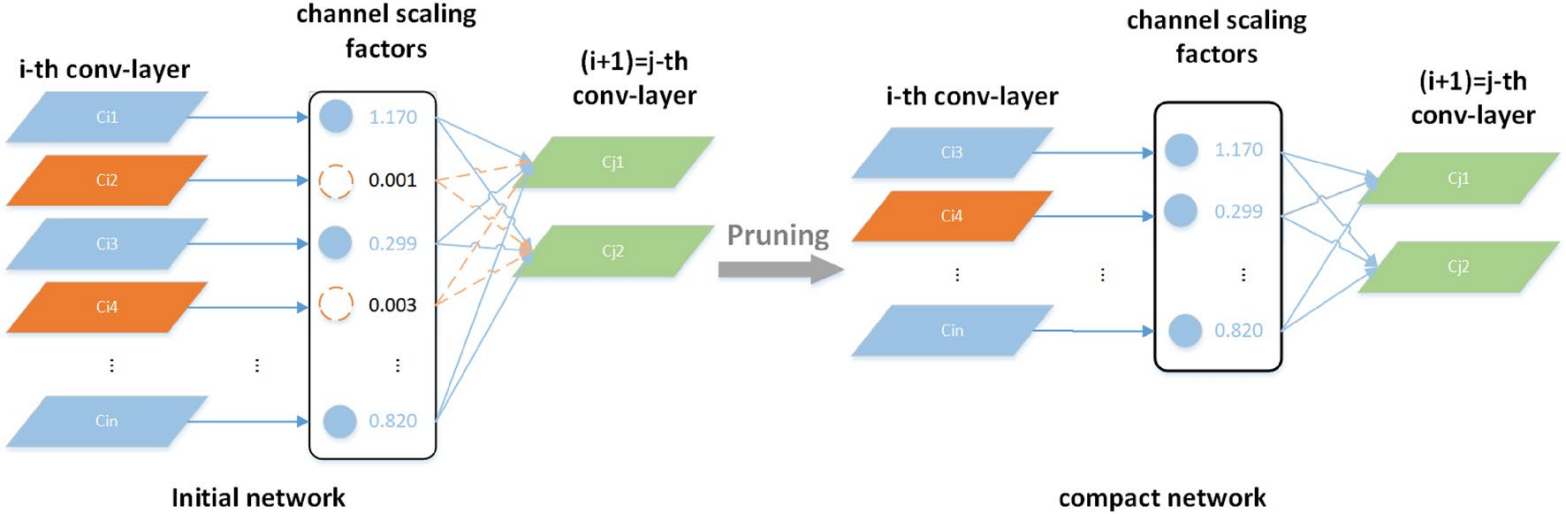
Schematic diagram of channel pruning.

After determining the sparsity rate to complete the sparsification, a scaling factor γ is used to measure the importance of the channel output. When γ is a small value, the C value obtained from Eq. (2) will be correspondingly small. The appropriate pruning rate is selected according to the effect of the model with different pruning ratios, and the channels with a negligible impact on these detection results (the orange part) are pruned to save the output of the channels with high importance, as schematically shown in Fig. 7.

**Figure 8:**
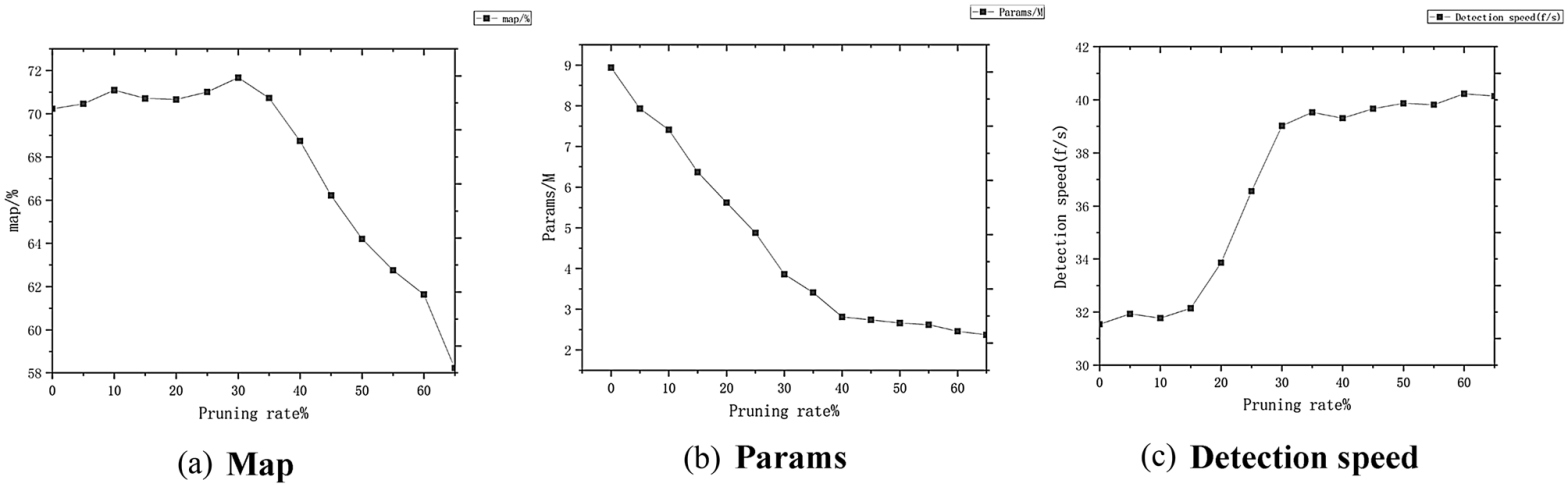
Variation of model parameters at different pruning rates.

In this paper, different pruning ratios are used to prune the YOLOX_S model that has completed the sparsification, and the gamma value during the pruning process suggests that the pruning rate in this paper takes value ranges from 0 to 0.6705. Figure 8 represents the variation of model parameters after fine-tuning at different pruning rates when the sparsity is taken as 0.005.

In Fig. 8a, when the pruning rate is less than 0.3, the model has a small range of variation in the map values; the map values were highest when the pruning rate was 0.3, the map value is reduced by 0.7% compared to regular training and indicates the best recognition; the map value decreases rapidly as the shear rate increases when the shear rate is more significant than 0.3. In Fig. 8b, the number of senators decreases as the clipping rate increases. In Fig. 8c, the detection speed of the original model is 32.24 fps, and the detection speed changes smoothly when the pruning rate is more significant than 0.3; the increase in detection speed is more significant when the pruning rate is more significant than 0.2. Combined with the change of model parameters, in order to ensure that the number of parameters is as tiny as possible and the detection speed is as large as possible under the conditions of the best choice of map value, the pruning rate in this paper is taken as 0.3, which can effectively reduce the number of model parameters to improve the detection speed and accuracy.

The pruned model network suffers from the reduced learning ability of the pruned part and reduced detection accuracy, thus requiring fine-tuning of the streamlined model. The fine-tuned model should enter the pruning module again to prune the next layer of channels, select the pruning rate and other parameters again by comparing the model effect, and iterate the above process until the detection model reaches the requirements of the optimization goal. The steps of the model pruning operation are as follows:

1. This experimental dataset is input into the YOLOX_S network for training to obtain an initial model;
2. Equations  (1) and (2) are used to normalize the channel parameters;
3. To determine the sparsity rate, the L1 regular term is added, and the BN layer coefficients are sparsified using Eqs. (3) and (4);
4. To extract the scaling factors of all channels and rank their absolute values, the model effects of different pruning rates are evaluated to determine the appropriate pruning rate and threshold, and channels below the threshold are pruned out, and those above the threshold are retained;
5. Refinement of the model;
6. The iterations of the above steps are repeated until the optimal result is achieved.

### Improvement of attention mechanisms

The human visual attention mechanism is the ability to select the interesting parts of an image for observation^32^. Suppose the feature extraction network also has an excellent ability to do this in target detection. In that case, it can reduce the interference of a large amount of redundant information caused by the convolution operation and then improve the network model's attention to small target features and the accuracy of small target detection.

**Figure 9:**
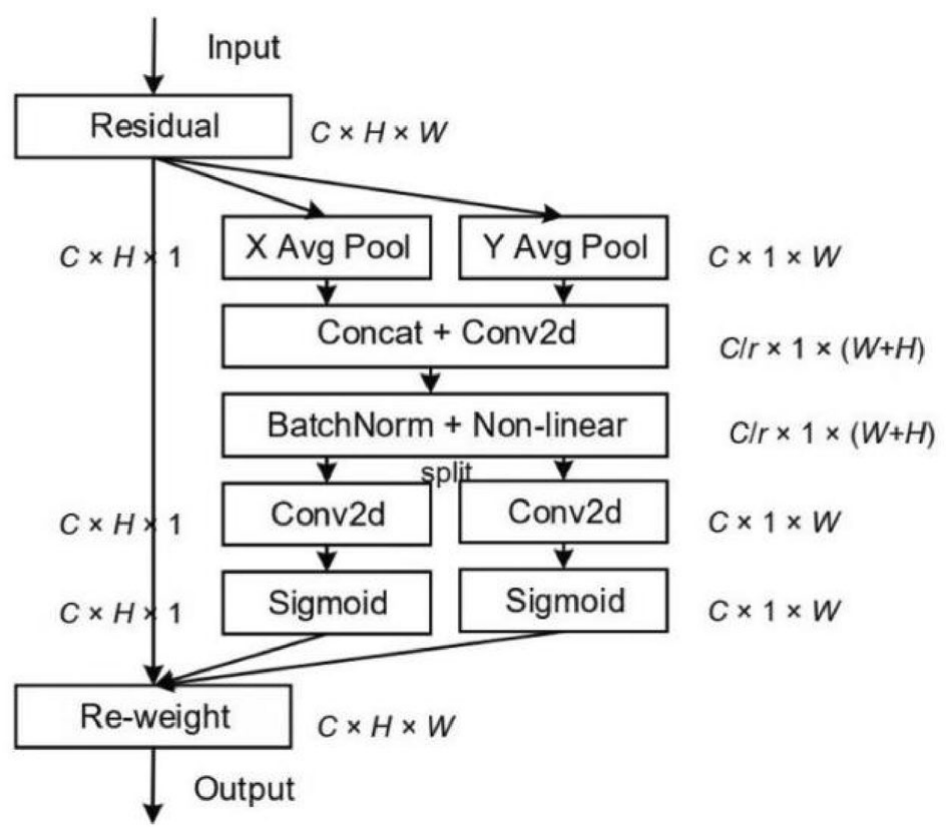
Schematic diagram of the CA module structure.

Attention is divided into spatial attention and channel attention^33^. Spatial attention is to obtain the weights of the two dimensions of height H and width W and multiply them by the feature area to obtain the feature information; channel attention is to obtain the weights of the channel dimension C and then multiply them by the feature area. The attention mechanisms commonly used in neural networks are SE, CAM, SAM, CBAM, and BAM modules. SE module (Squeeze-and-Excitation Networks) is a lightweight attention module that obtains the importance degree of different channels through the global average pooling operation and the two fully connected layers and uses this importance degree to give different weight values to each feature with different weight values to obtain more important feature information, but does not consider the attention of obtaining location information; CAM Module (Channel Attention Module) means the channel attention module, which attaches importance to the information of each channel of the feature map, and uses the average pooling and maximum pooling to compress the spatial information in conjunction with the input image, and finally obtains the channel attention feature map. SAM module (Spatial Attention Module) is the spatial attention module that attaches importance to the location information of the features and selectively focuses on each spatial feature information by weighting the spatial features; CBAM module (Convolutional Block Attention Module) represents the lightweight convolutional attention module, contains CAM and SAM sub-modules for channel and spatial attention extraction respectively, and the outputs of the two sub-modules are multiplied to obtain the attention vector; BAM module (Bottleneck Attention Module) denotes the bottleneck attention module, which can be connected to any feed-forward convolutional neural network and infer the attention mapping along the two paths of channel and space. CBAM can be seen as a tandem connection between CAM and SAM, and BAM can be seen as a parallel connection between the two. However, BAM and CBAM separate spatial and channel attention and can only obtain local range information, not the remote interaction relationship of feature maps. The CA module refers to the coordinate attention module, which embeds the spatial position information into the channel attention and comprehensively considers the relationship between the channels and the spatial position information of the features, which can alleviate the problem of fuzzy or missing information of the deep feature edges of the small target, and improve the localization ability of the feature information in the target area. Zha et al.^34^ embedded the coordinate attention module into the lightweight feature extraction module of the YOLOv4_tiny model to enhance the feature information, and the experimental results showed that the improved method improved the detection model detection; Zhang et al.^35^ introduced the coordinate attention module into the tail of the backbone network of the YOLOv5, which improved the detection accuracy of the model. The structure of the CA module is shown in Fig. 9.

The specific operation of the coordinate attention module is divided into two steps: coordinate information embedding and coordinate attention generation. In the coordinate information embedding session, the CA module first performs a global average pooling operation on the input features (C × H × W) in the horizontal and vertical directions sequentially to obtain two feature maps of C × H × 1 and C × 1 × W. The global average pooling calculation formula is as follows:

5 $$z_{c} = \frac{1}{H \times W}\sum\limits_{i = 1}^{H} {\sum\limits_{j = 1}^{W} {x_{c} (i,j)} }$$

In the formula, C is the number of input feature channels; H is the height of the input feature; W is the width of the input feature; Z_c_ is the output feature of channel c; and x_c_ is the input feature of channel c.

Specifically, each channel is horizontally and vertically coded using pooling kernels of dimensions (H,1) and (1, W), respectively, to obtain two outputs of height h and width w in channel c, calculated as follows:

6 $$T_{c}^{h} (h) = \frac{1}{W}\sum\limits_{0 \le i < W} {x_{c} (h,i)}$$

7 $$T_{c}^{w} (w) = \frac{1}{H}\sum\limits_{0 \le j < H} {x_{c} (j,w)}$$

In the formula, T^h^_c_(h) is the characteristic output of channel c in the vertical direction; T^w^_c_(w) is the characteristic output of channel c in the horizontal direction;

After the above two transformations, the distance dependency in one spatial direction and the positional accuracy information in another can be obtained, completing the embedding of the coordinate information. The two output features obtained in the previous step are then subjected to a splice operation, and the channel is compressed using a 1 × 1 convolution; after BN normalization and non-linear activation function, the intermediate features are separated and decomposed into two directional feature tensors f^h^ and f^w^, and then the two feature tensors are subjected to 1 × 1 convolution operation to adjust the number of channels as well as sigmoid activation operation to obtain the two components g^h^ and g^w^, which are consistent with the number of channels of the input features, and the calculation formulae are as follows:

8 $$f = \delta \left( {F_{1} ([z^{h} ,z^{w} ])} \right)$$

9 $$\left\{ \begin{gathered} g^{h} = \sigma (F_{h} (f^{h} )) \hfill \\ g^{w} = \sigma (F_{w} (f^{w} )) \hfill \\ \end{gathered} \right.$$

In the formula, f is the intermediate feature map generated by the position information in two spatial directions; $\delta$(·) is the non-linear activation function; $\sigma$(·) is the sigmoid activation function; and F(·) are all convolution functions.

Finally, the two components g^h^ and g^w^ are weighted with the original input features to obtain the final output y_c_ of the CA module in channel c. The formula is as follows:

10 $$y_{c} (i,j) = x_{c} (i,j) \times g_{c}^{h} (i) \times g_{c}^{w} (j)$$

In the formula g^h^_c_(i) and g^w^_c_(j) are the weight values in different directions.

**Figure 10:**
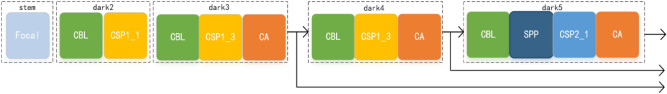
Backbone_CA structure.

**Figure 11:**
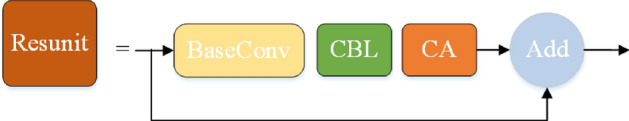
Resunit_CA structure.

The Coordinate Attention Module has plug-and-play characteristics and is deployed in the backbone network to enhance the feature extraction capability of the backbone network. In order to choose the appropriate location for the CA module addition, this paper adds the CA module to the three adequate feature layer tails and residual structures of the backbone network of the YOLOX_S model for experimental performance comparison, respectively, and the improved structure are shown in Figs. 10 and 11.

**Figure 12:**
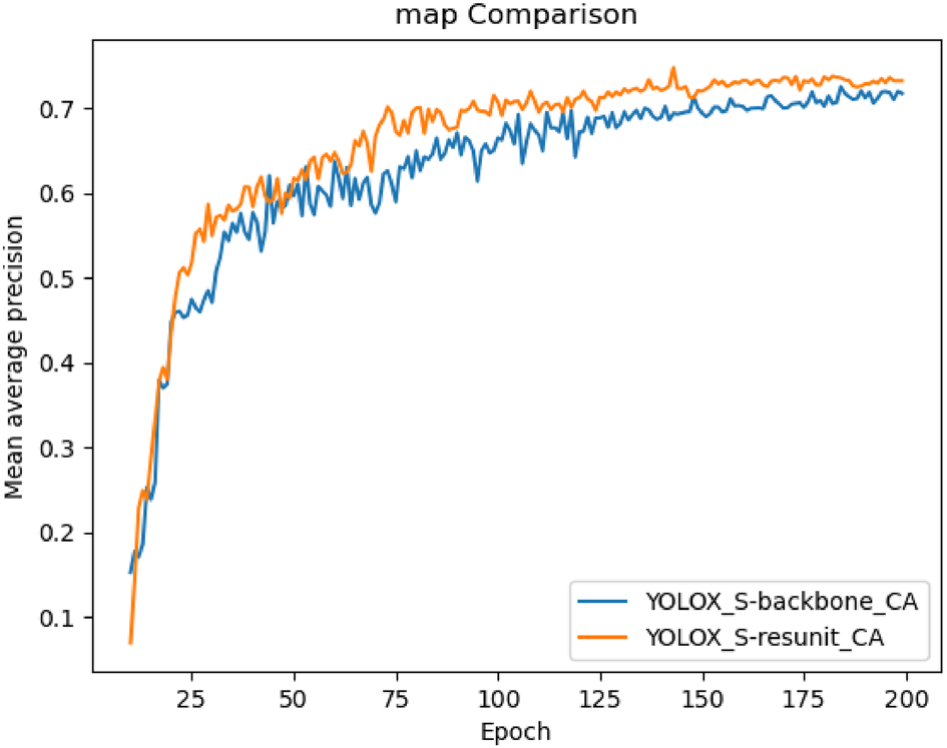
map curve comparison.

The CA modules were added in two locations to train 200 rounds on this experimental dataset to obtain the improved models YOLOX_S-backbone_CA and YOLOX_S-resunit_CA. The results of the comparison of the two model training MAP curves are shown in Fig. 12.

Based on the pruned YOLOX_S model, the detection performances of adding the CA module to the three effective feature layers in the feature extraction network, the residual structure, and the detection model added at both of these positions are compared. Among them, the residual structure is added at the position after the convolution operation and the CBL structure. The feature information obtained from the Focus structure is entered into the dark2, dark3, dark4, and dark5 modules, respectively, and the CSP1_x structure can be improved to improve the learning ability of the small target head features by the improved residual structure; the CA module added in the effective feature layer is located behind the CSP1_3 and CSP2_1 structures in the dark3, dark4, and dark5 modules, with the extracted features of three scale sizes of 80 × 80 × 256, 40 × 40 × 512 and 20 × 20 × 1024 being processed by the CA module to input the feature information into the enhanced feature extraction network for further deep feature extraction.

From Fig. 12 and Table 1, it can be seen that adding the coordinate attention module to the residual structure improves the model's mAP by 0.92%, adding it to the three effective feature levels improves the mAP by 0.38%, and adding both positions to the CA module at the same time improves the model’s mAP by 0.13%. Since the increase in the convolution operation resulted in a slight increase in the number of model parameters, and the addition of the CA module at a single location did not result in an increase in model computation, and therefore the detection accuracy and speed of the model was inferior to that of the model with the CA module introduced at two locations. The results show that the degree of influence on the model detection performance is different when the coordinate attention module is added at different positions of the model. The model map value obtained by adding the CA module to the residual structure is better than that obtained by adding it to the effective feature layer, so the CA module is considered to be added to the residual structure from the aspects of detection accuracy and detection speed.

**Table 1:** Comparison of experimental performance at different addition locations.

Placement	mAP/%	Params/M
No additions	72.33&	3.86
Residual structure	73.25%	3.86
Effective feature layer	72.71%	3.86
All	72.46%	3.88

### Adaptively spatial feature fusion module(ASFF)

**Figure 13:**
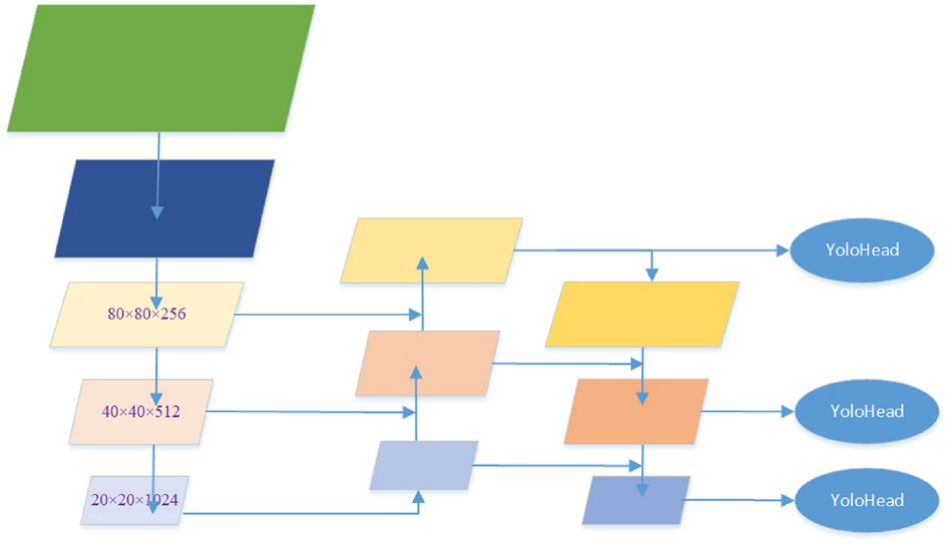
PAFPN structure of YOLOX_S.

The PAFPN structure is mainly composed of feature pyramid network FPN and path aggregation network PAN structure and usually uses splicing or pixel fusion and other direct articulation or summation to complete the fusion of deep features and shallow features, which can not make full use of the feature information of different feature scales between the multi-layers, and the richness of features can be extracted is limited^36^. Its structure is shown in Fig. 13.

**Figure 14:**
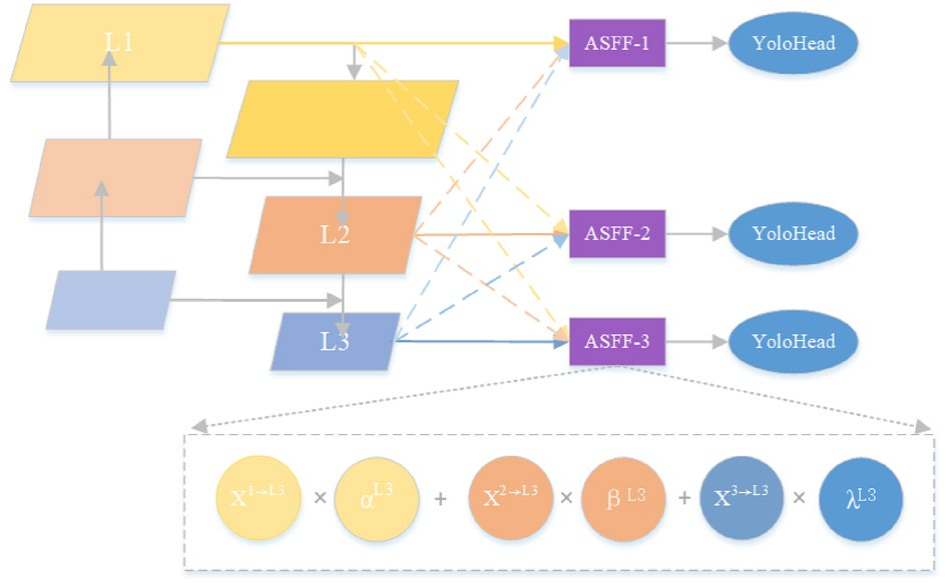
PAFPN + ASFF structure.

In the process of target detection, large targets require larger sensory fields and deeper semantic features to be more easily captured by deeper features. In contrast, recognizing small target features requires more shallow feature localization information to play a role. The core idea of the adaptively spatial feature fusion module ASFF is to multiply and then fuse (sum) the three scale layer features L1–L3 generated by PAFPN with the corresponding learnable weights to obtain three corresponding scale features ASFF-n, n = 1,2,3, and its structure is shown in Fig. 14.

Taking ASFF-3 in the figure as an example, L_1_–L_3_ are the three enhanced feature layers extracted by the PAFPN structure, whose features are denoted as X^1^, X^2^, and X^3^, respectively. Firstly, the number of feature channels of the three scales is adjusted to be equal to the number of channels of L_3_ by the operation of up-sampling or down-sampling. Then, the fusion weight parameters α, β and $\lambda$ are learned and defined using the softmax with the control scalars $\lambda_{\alpha }$, $\lambda_{\beta }$ and $\lambda_{\gamma }$ functions, respectively. The calculation formula is as follows:

11 $$\left\{ \begin{gathered} \alpha_{ij}^{{L_{n} }} = \frac{{e^{{\lambda_{{\alpha_{ij} }}^{{L_{n} }} }} }}{{e^{{\lambda_{{\alpha_{ij} }}^{{L_{n} }} }} + e^{{\lambda_{{\beta_{ij} }}^{{L_{n} }} }} + e^{{\lambda_{{\gamma_{ij} }}^{{L_{n} }} }} }}; \hfill \\ \beta_{ij}^{{L_{n} }} = \frac{{e^{{\lambda_{{\beta_{ij} }}^{{L_{n} }} }} }}{{e^{{\lambda_{{\alpha_{ij} }}^{{L_{n} }} }} + e^{{\lambda_{{\beta_{ij} }}^{{L_{n} }} }} + e^{{\lambda_{{\gamma_{ij} }}^{{L_{n} }} }} }}; \hfill \\ \gamma_{ij}^{{L_{n} }} = \frac{{e^{{\lambda_{{\gamma_{ij} }}^{{L_{n} }} }} }}{{e^{{\lambda_{{\alpha_{ij} }}^{{L_{n} }} }} + e^{{\lambda_{{\beta_{ij} }}^{{L_{n} }} }} + e^{{\lambda_{{\gamma_{ij} }}^{{L_{n} }} }} }}; \hfill \\ \end{gathered} \right.$$

The weight parameters need to be satisfy: $\alpha_{ij}^{{L_{n} }} + \beta_{ij}^{{L_{n} }} + \gamma_{ij}^{{L_{n} }} = 1;\alpha_{ij}^{{L_{n} }} ,\beta_{ij}^{{L_{n} }} ,\gamma_{ij}^{{L_{n} }} \in [0,1][0,1].$

In the formula, i and j denote rows and columns, respectively; parameters $\lambda_{\alpha }$, $\lambda_{\beta }$ and $\lambda_{\gamma }$ are obtained from X^1^, X^2^, and X^3^ after 1 × 1 convolution, respectively, and can be learned by backpropagation until the appropriate weights are found.

The last three features with the same number of channels are multiplied and summed with the corresponding weights to obtain the new feature ASFF-3 ($y_{ij}^{{L_{n} }}$), which is calculated as follows:

12 $$y_{ij}^{{L_{n} }} = \alpha_{ij}^{{L_{n} }} \cdot X_{ij}^{{1 \to L_{n} }} + \beta_{ij}^{{L_{n} }} \cdot X_{ij}^{{2 \to L_{n} }} + \gamma_{ij}^{{L_{n} }} \cdot X_{ij}^{{3 \to L_{n} }}$$

In order to perceive and detect the detailed features of small target vehicles, it is necessary to make full use of the semantic information of the deep features and the positional information of the shallow features for strengthening the learning ability of the fine-grained features in the shallow features. In this paper, the ASFF module is added to the tail of the PAFPN structure to multiply and then add the features of each layer with its corresponding weight parameters to achieve fusion. The proportion of features in each layer is adjusted using the parameters, which can effectively retain the features in the region of interest and filter the unimportant feature information so that the features extracted by the model are more hierarchical to improve the model's ability to learn the features of small targets.

### Loss function improvements

A loss function is used in the prediction phase of the model to measure the extent to which the predicted value differs from the actual value. The larger the error is, the larger the loss value. During the iterative training of the network model, the loss value is continuously minimized, and the loss value can be used as a reference for backpropagation to update the model parameters to bring the predicted value closer to the actual value^37^. In the YOLOX model, after completing the third part of the refined allocation strategy, the error between the actual frame and the predicted frame is calculated to obtain the loss function value. The loss function for the YOLOX model consists of category prediction loss (loss_cls)—using binary cross-entropy loss, object existence probability loss (loss_obj)—using binary cross-entropy loss, and IOU loss (loss_iou)—using IOU cross-merge ratio.

The YOLOX model does not need to generate candidate frames to directly associate the predicted and labeled frames, which improves the speed of model inference but also results in an imbalance of positive and negative samples. There are more candidate frames without targets than with targets, resulting in decreased model accuracy. In actual practice, the different control requirements for the type and number of vehicles in operation in each traffic scenario often result in more cars than other types of vehicles and the presence of more difficult-to-classify samples, leading to unstable model performance. The presence of occlusion can also cause problems with small targets being inconspicuous or misdetecting traffic devices on the road as positive samples, and these problems will increase the difficulty of tracking the trajectories of small cars in future traffic accident detection studies. Liu et al.^38^ changed the cross-entropy loss function of YOLOv3_tiny to Focal Loss loss function and conducted comparison experiments with the original loss function, and the results showed that the detection accuracy of the model using Focal Loss was 4.25% higher compared to the original model; Zhang et al.^39^, to solve the problem of imbalance between the positive and negative samples and the difficult and easy samples, replaced the classification loss function of YOLOv5 to Focal Loss loss function, and the detection accuracy reaches 92.5%. In order to improve the problem of detection accuracy degradation caused by the uneven number of positive and negative samples, this paper studies the classification prediction part on the three decoupling head branches. The binary cross-entropy function BCEWithLogitsLoss for the category prediction loss is replaced by the focal loss function^12^ to improve the model’s focus on difficult-to-classify or small target samples.

The binary cross-entropy loss function is calculated as follows:

13 $$CE(p,y) = - y\log p - (1 - y)\log (1 - p) = \left\{ {\begin{array}{*{20}c} { - \log (p)} & {if{\text{ y}} = 1} \\ { - \log (1 - p)} & {otherwise} \\ \end{array} } \right.$$

In the formula, y = 1 is the positive sample label value; otherwise, it represents a negative sample; *p* $\in { }$[0,1] is the probability that the model predicts y = 1, and the larger the *p*-value indicates that the model is more accurate in its prediction, so BCELoss is not able to balance the positive and negative categorization of samples well. By defining p_t_ as: $$p_{t} = \left\{ {\begin{array}{*{20}c} p & {if\;y = 1} \\ {1 - p} & {otherwise,} \\ \end{array} } \right.$$ and obtaining the following formula:

14 $$CE(p,y) = CE(p_{t} ) = - \log (p_{t} )$$

Among these, the p_t_ can directly reflect the proximity of the predicted classification to the positive sample, and the larger p_t_ indicates the more accurate classification results. A hyperparameter α is introduced to balance the proportion of positive and negative samples based on Eq. (14), and a parameter (1 − p_t_)^γ^ is set to increase the attention to hard-to-classify samples to obtain the formula for calculating the Focal loss function:

15 $$FL(p_{t} ) = - \alpha_{t} (1 - p_{t} )^{\gamma } \log (p_{t} )$$

16 $$FL(p) = \left\{ {\begin{array}{*{20}c} { - \alpha (1 - p)^{\gamma } \log (p)} & {if\;y = 1} \\ { - (1 - \alpha )p^{\gamma } \log (1 - p)} & {otherwise} \\ \end{array} } \right.$$

In the formula, γ $\in$ [0,5] is used to regulate the weights of positive and negative samples; α $\in$ [0,1] is a weight factor to balance the classification of positive and negative samples.

Based on the above improvement method, this paper improves the loss function of the Head structure of the optimized YOLOX_S model. The parameter γ is set to 2 when the weight factor α is set to 0.35 to alleviate the uneven classification of samples.

## Experiments and analysis of results

### Experimental platform and parameter configuration

The parameters of the equipment used in the experiments of this paper are shown in Table 2:

**Table 2:** Experimental equipment parameter table.

Hardware and software	Version model
Computer system	Windows 10
Computer processor	Intel(R) Core(TM) i5-8250U CPU @ 1.60 GHz 1.80 GHz
Operational memory	8 GB
GPU	NVIDIA GeForce 940MX
Graphics memory	4 GB
Experimental software	PyCharm2021.3.1
PyTorch	1.13.1
Language	Python3.8
Configuration device	NVIDIA CUDA11.6 Cudnn8.4.0

The experimental training parameters and formula parameter settings are shown in Table 3:

**Table 3:** Table of training parameters and formula parameters.

Parameter name	Parameter value
Epochs	200
batch_size	8
Image size	640 × 640
Sparsity rate	0.005
Pruning rate	0.3
Weight factor-α	0.35
γ	2

### Datasets and evaluation indicators

The UA-DETRAC^40^ dataset is a publicly available vehicle detection dataset with videos from 24 locations in Beijing and Tianjin, providing tens of thousands of images with annotations for vehicle detection categories. The dataset has a rich set of traffic scenes and was captured under different weather conditions and light intensities, including four categories: cars, buses, trucks, and others.

**Figure 15:**
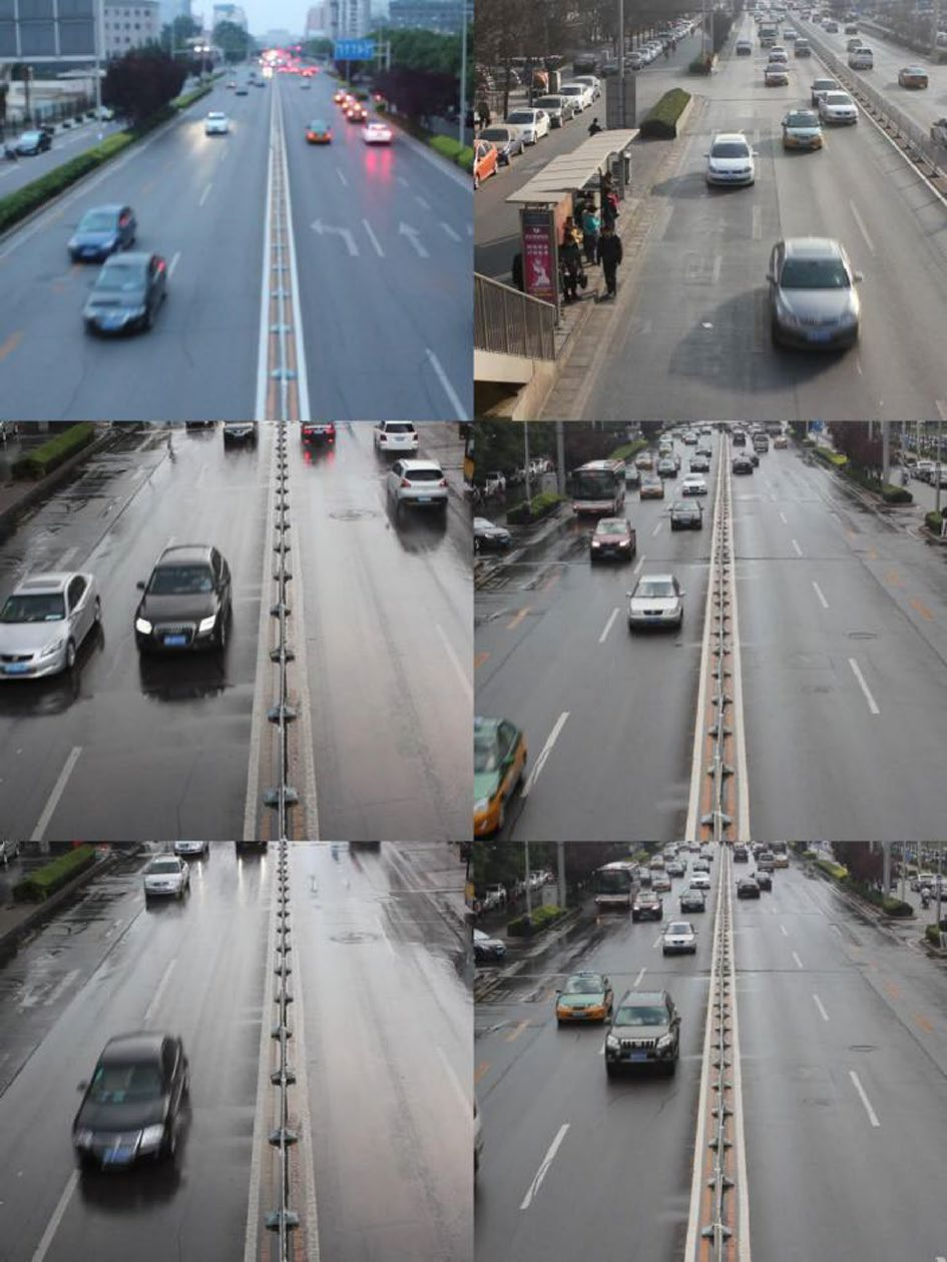
Partial pictures of selected datasets.

**Figure 16:**
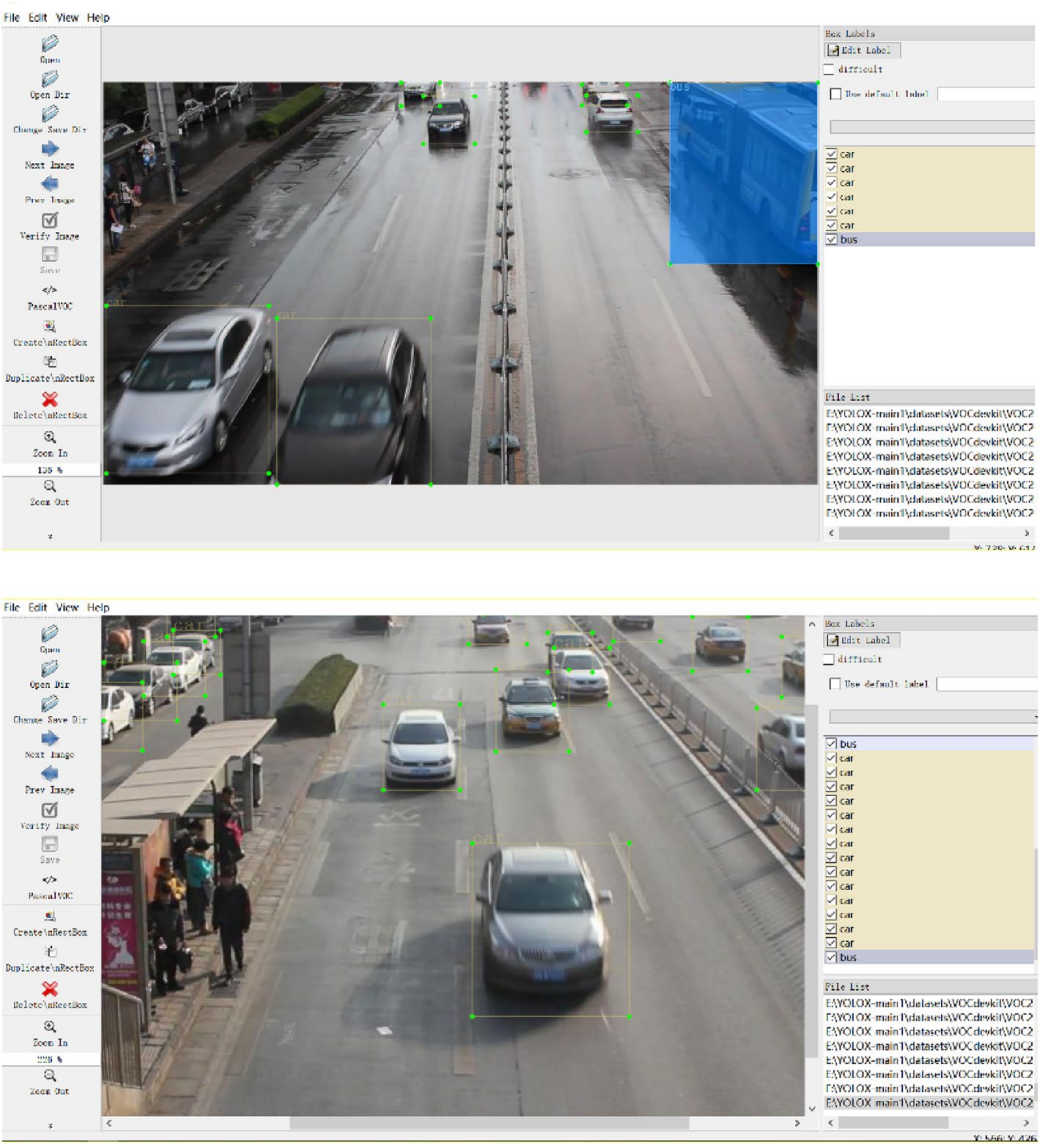
LabelImg interface.

The experimental dataset in this paper is selected from some video sequences in MVI_20011, MVI_39761, MVI_40131, and MVI_63521 of the UA-DETRAC public vehicle detection dataset, with a total of 4570 video frame screenshots taken at a resolution of 960 * 540 pixels, and the dataset sample is shown in Fig. 15. Considering that the traffic scenarios in the dataset samples are mainly urban roads, and the restrictive driving policies for trucks are set in several administrative areas in Beijing and Tianjin, the number of car samples in the dataset samples is the largest, minivans and buses are smaller, and the number of trucks is only a few. To avoid the problem of unbalanced sample size and overfitting of the model, this paper does not include trucks in the labeling categories. It classifies buses and minivans into the same category, and the detection targets into two categories, car and bus. The annotation software Labelimg is used to re-label the selected images into categories, generate the dataset in Pascal VOC format, and divide the dataset into training, validation, and test sets in the ratio of 8:1:1. Labelimg annotation interface is shown in Fig. 16. The .xml file records the location information, and each line represents a target, which is separated by a space, and represents the category ID of the target, and the x coordinate of the centroid and y coordinate of the centroid after the normalization process, w and h of the target box, respectively.

In this paper, to validate the performance of the improved model, ablation experiments, comparison experiments of different detection models, and multiple metrics were used to evaluate the optimization of the improved detection model. The evaluation metrics used in this experiment are Average Precision (AP), Mean Average Precision (mAP), and Frame Per Second (FPS).

TP (True Positives) indicates the number of predicted positive samples that are actually positive samples;

FP (False Positives) indicates the number of samples that are predicted to be positive and are actually negative samples;

TN (True Negatives) indicates the number of predicted negative samples that are actually negative samples;

FN (False Negatives) indicates the number of predicted negative samples that are actually positive samples.

17 $${\text{P}} = {\text{Precision}} = \frac{{{\text{TP}}}}{{{\text{TP}} + {\text{FP}}}}$$

18 $$R = {\text{Re}} call = \frac{TP}{{TP + FN}}$$

19 $$AP = \int_{0}^{1} {P(R)d(R)}$$

20 $$mAP = \frac{1}{C}\sum\limits_{i = 1}^{C} {AP(i)}$$

In the formula, Precision is the accuracy rate, which indicates the probability that the predicted samples are positive samples; Recall is the recall rate, which indicates the probability that the positive samples are correctly identified.

### Analysis of experimental results

**Figure 17:**
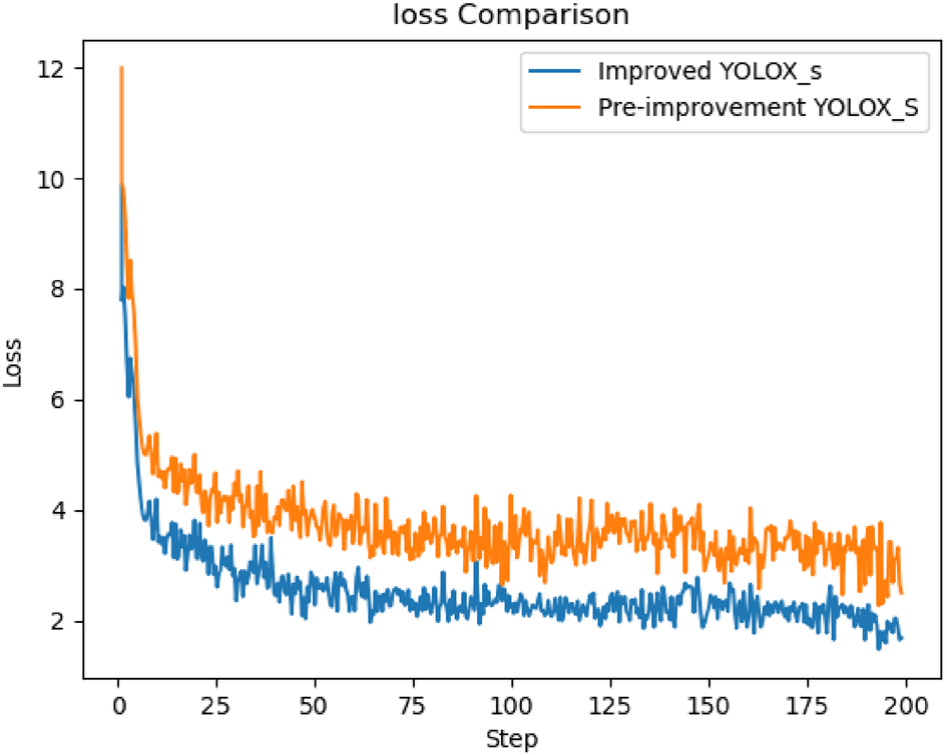
Comparison of model loss curves before and after improvement.

Comparing the training loss curves before and after the improvement of the YOLOX_S model, it can be observed from Fig. 17 that the loss value decreases rapidly in the pre-training period. However, as the number of training rounds increases, after about 25 rounds, the decreasing trend of the loss value is smooth, and after training to 75 rounds, the loss value decreases slowly. The optimized model loss curve has a lower loss value than the original model loss curve, and the convergence is smoother and faster, so the improved method and each experimental parameter setting in this paper help improve the detection accuracy.

In order to verify the effectiveness of the improved model in this paper, an ablation experiment is designed, and the influence of different improvement methods on the original YOLOX_S model is shown in Table 4. “ × ” indicates that the method was not used, and “√” indicates that the method was used. The experimental results showed that the feature extraction ability was weakened after pruning the original YOLOX_S model. However, the accuracy was only reduced by 0.7%, and the FPS was improved from 32 to 44 fps. The reason for this was the streamlining of the baseline network structure and the reduction in the number of model parameters, which improved the detection speed. It shows that the model pruning method can improve the detection speed while maintaining the detection accuracy for this dataset. Based on the pruning of the YOLOX_S model, the original residual structure is optimized and improved to obtain the resunit_CA structure, with the mAP value improved by 1.58%, but the detection speed reduced to 41 fps due to the increase in the number of convolutional operations. After introducing the adaptively spatial feature fusion ASFF, the increase in the number of model parameters led to a decrease in the detection speed of 10.56 fps. However, the effect of detection accuracy enhancement was evident, and the mAP value was improved by 3.07%. It shows that ASFF makes fuller use of multi-scale feature layers and effectively strengthens the model's ability to extract features from small targets. Optimizing the loss function based on the first three improved methods and using the Focal Loss function to regulate the distribution of positive and negative samples, the mAP was further improved by 0.87%, and the detection speed was reduced to 29 fps. The reduced detection speed (increased inference time) of the improved YOLOX_S model compared to the original YOLOX_S model was mainly due to the increased resolution of the input feature maps.

**Table 4:** Ablation experiments.

Model	Prune	Coordinate attention	ASFF	Focal loss	mAP/%	FPS(f/s)	Params/M
YOLOX_S	×	×	×	×	0.7233	32.24	8.94
YOLOX_S-prune	√	×	×	×	0.7167	44.07	3.86
YOLOX_S-prune + CA	√	√	×	×	0.7325	41.31	4.27
YOLOX_S-prune + CA + ASFF	√	√	√	×	0.7632	31.87	9.75
YOLOX_S-prune + CA + ASFF + Focal Loss	√	√	√	√	0.7719	29.73	9.68

Contrasting experiments are conducted on the dataset of this paper using the two-stage target detection model SSD and the one-stage target detection models YOLOv3, YOLOv4, YOLOv5_s, as well as the original YOLOX_S model with the improved model, and the results of the experimental performance of the different models are shown in Table 5. The results of the metrics, such as AP value, mAP value, and detection speed FPS for each category of the improved model, are better than the SSD, YOLOv3, YOLOv4, and YOLOv5_s models. From the experimental data, the detection accuracy of the improved model is completely better than that of the original YOLOX_s model, which is valuable in terms of detection effect.

**Table 5:** Contrast experiment.

Target detection model	AP/%		mAP/%	FPS/(f/s)
	Car (%)	Bus (%)		
SSD	45.97	38.29	42.13	7.41
Faster-RCNN-VGG16	61.79	52.37	57.08	5.39
Faster-RCNN-Res-101	63.48	58.94	61.21	2.76
YOLOv3	62.42	54.56	58.49	14.82
YOLOv4	71.39	59.85	65.62	20.14
YOLOv5_s	76.47	63.21	69.84	24.23
The original YOLOX_S	80.23	64.42	72.33	32.24
The improved YOLOX_S	84.16	70.22	77.19	29.73

The comparison of the performance parameters of different detection models shows that in terms of detection accuracy, the improved model in this paper improved 35.06%, 20.11%, 15.98%, 16.1%, 8.97%, 4.75%, and 4.86% over SSD, Faster-RCNN-VGG16, Faster-RCNN-Res-101, YOLOv3, YOLOv4, YOLOv5_s, and the original YOLOX, respectively; The AP values of each category of the improved YOLOX_S model were higher than those of the other models, and the AP values of the car and bus categories of the improved model were respectively improved by 3.93% and 5.8% compared to the original YOLOX_s model; In terms of detection speed, the improved model was 22.32 fps, 24.34 fps, 26.97 fps, 14.91 fps, 9.59 fps, and 5.5 fps faster than the SSD, YOLOv3, YOLOv4, and YOLOv5_s models respectively, but inferior to the original YOLOX_S model. However, from the overall data, the improved YOLOX_S model possesses higher detection accuracy and real-time performance than the SSD, Faster-RCNN-VGG16, Faster-RCNN-Res-101, YOLOv3, YOLOv4, and YOLOv5_s models, which have certain advantages.

**Figure 18:**
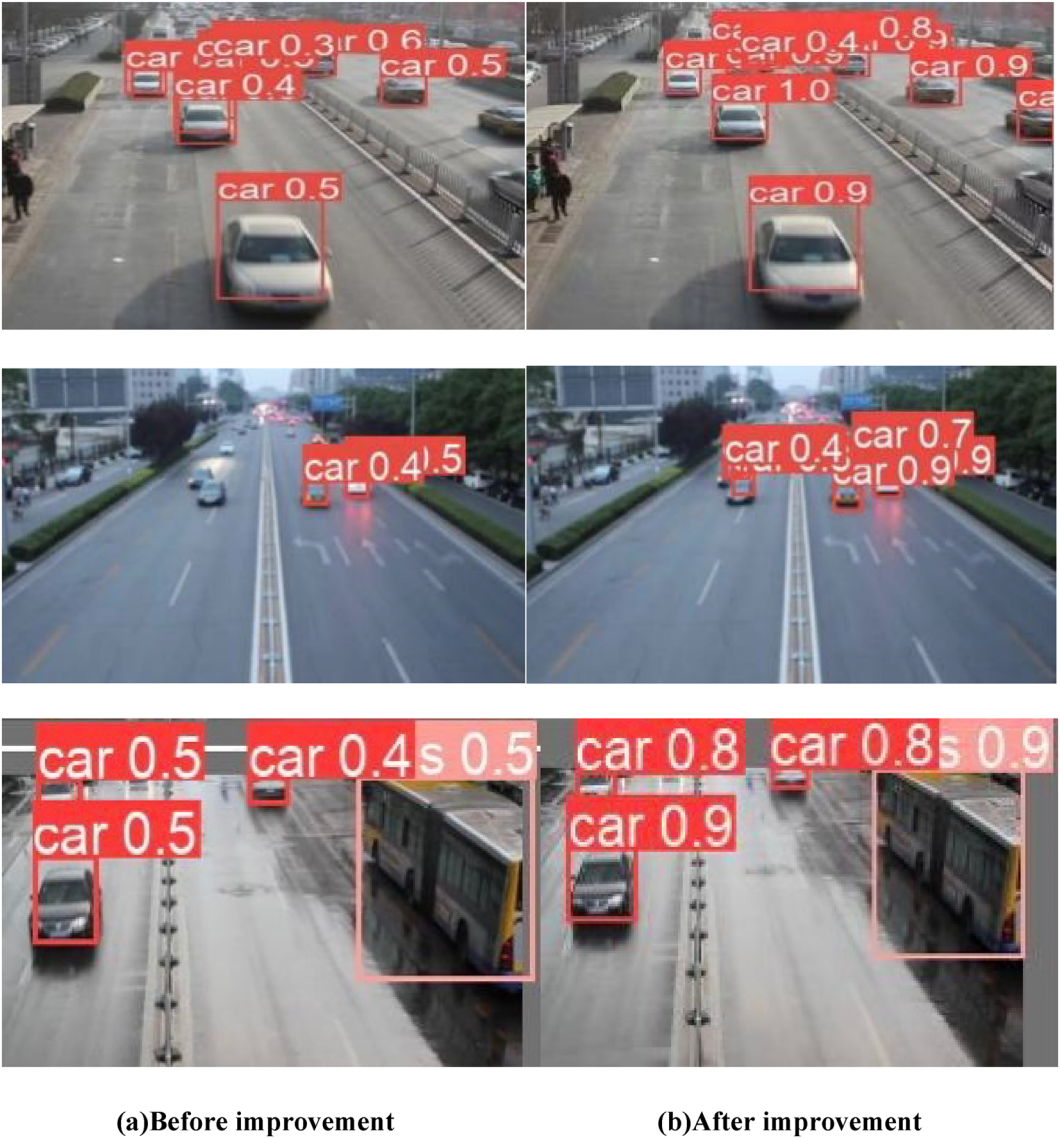
Comparison of model detection effect before and after improvement.

The original YOLOX_S model and the improved model are used to visualize vehicle detection in three traffic scenarios, and the results are shown in Fig. 18. The left column of images (a) shows the original YOLOX_S detection results, and the right column of images (b) shows the detection results of the improved model. As can be seen from the comparison graph, the improved model can detect long-range small targets not detected by the original YOLOX_S model in low-light environments, and the detection accuracy of small targets was also better than that of the original YOLOX_s model. Compared with the original YOLOX_S model, the improved model was more effective in detecting target vehicles at the boundary of the surveillance range. According to the visualization detection effect, the improved model detection effect is better than the original YOLOX_s model, and it can more accurately identify and detect vehicle targets in different light and distance, alleviating the problems of multi-target occlusion and small targets that are not easy to identify. Therefore, the effectiveness and real-time performance of the optimized and improved model of this paper for vehicle detection in traffic scenarios are further verified.

## Conclusions

Aiming at the problem of multi-target and micro-target detection in traffic scenarios, this paper was based on the original YOLOX_S target detection model, and through the model pruning operation, the number of model parameters was reduced, and the detection speed was greatly improved; to compensate for the decrease in model detection accuracy caused by the pruning operation, the coordinate attention module was added to the effective feature layer and the residual network respectively and trained and tested on the dataset, and attention module was selected to be added to the residual structure according to the average accuracy rate of the detection, and the detection performance of the model was optimized; to make the model extract richer feature information, this paper added the adaptively spatial feature fusion ASFF to the tail of the PAFPN structure for deep features and shallow features, and the detection accuracy was significantly improved; optimizing the loss function in the detection header of the prediction layer improved feature learnability by increasing the weights of complex samples and controlling the loss values of positive and negative samples. The experimental results show that compared with the original YOLOX_S detection model and some of the current mainstream models, the improved model proposed in this paper effectively improved the accuracy of the detection precision, and the detection precision reached 77.19%. However, the detection speed decreased to 29.73 fps. In the subsequent research, real-time vehicle detection will be further optimized to provide a basis for real-time detection of traffic accidents in traffic management in the future.

## Data Availability

The public dataset the UA-DETRAC used in this research can be found at the following link: https://detrac-db.rit.albany.edu/. The source code of the algorithms used in this research can be found in the Github repositories [https://github.com/Megvii-BaseDetection/YOLOX; https://github.com/ultralytics/yolov5; https://github.com/ Tianxiaomo/pytorch-YOLOv4.git; https://github.com/ultralytics/yolov3/tree/archive; https://github.com/amdegroot/ssd.pytorch]; https://github.com/jwyang/faster-rcnn.pytorch/tree/pytorch-1.0.
